# Posttraumatic growth and its association with unmet supportive care needs and fear of cancer progression among head and neck cancer patients

**DOI:** 10.1371/journal.pone.0265502

**Published:** 2022-03-15

**Authors:** Nik Ruzyanei Nik Jaafar, Nur Amirah Hamdan, Norhaliza Abd Hamid, Rama Krsna Rajandram, Raynuha Mahadevan, Hazli Zakaria, Mohd Razif Mohamad Yunus, Mohammad Farris Iman Leong Bin Abdullah

**Affiliations:** 1 Department of Psychiatry, Universiti Kebangsaan Malaysia Medical Centre, Cheras, Kuala Lumpur, Malaysia; 2 Department of Community Health, Advanced Medical and Dental Institute, Universiti Sains Malaysia, Kepala Batas, Pulau Pinang, Malaysia; 3 Department of Oral and Maxillofacial Surgery, Universiti Kebangsaan Malaysia Medical Centre, Cheras, Kuala Lumpur, Malaysia; 4 Department of Otorhinolaryngology, Universiti Kebangsaan Malaysia Medical Centre, Cheras, Kuala Lumpur, Malaysia; University Hospital Zurich, SWITZERLAND

## Abstract

**Background and aims:**

The assessment of supportive care needs and fear of cancer progression are important variables to be considered when evaluating the psychological aspects of cancer patients. However, data on how these variables affect posttraumatic growth (PTG) are lacking. This study aimed to investigate the level of PTG among head and neck cancer (HNC) patients within the first year of cancer diagnosis and to determine the association between unmet supportive care needs, fear of cancer progression, and the level of PTG.

**Methods:**

Participants were administered socio-demographic and clinical characteristics questionnaire; the 34-item Supportive Care Needs Survey (SCNS-34) to measure the unmet needs; 12-item Fear of Progression Questionnaire-Short Form (FoP-Q-SF) to measure the fear of progression of cancer; and the Posttraumatic Growth Inventory—Short Form (PTGI-SF) to measure the degree of PTG.

**Results:**

A total of 190 HNC participants reported a mean total PTGI-SF score of 39.3 (standard deviation = 9.5). General linear model revealed that higher degree of patients’ physical and daily living unmet needs and fear of cancer progression significantly predicted lower PTG, after controlling for sociodemographic and clinical factors.

**Conclusion:**

HNC patients within the first year of cancer diagnosis reported a high level of PTG. Despite that, psychosocial intervention for HNC patients should emphasize on counteracting patients’ physical and daily living unmet needs and fear of cancer progression to improve the psychological well-being of patients.

## Introduction

Head and neck cancer (HNC) is a group of biologically similar cancer diagnoses which affect the pharynx, larynx, nasal cavity, paranasal sinuses, oral cavity, cheek, and lips. In the Malaysian context, 9,419 cases of HNC were reported between 2007 and 2011, which made up of 9.1% of all cancer diagnoses rendering HNC as the fourth most prevalent cancer in the country [1]. In the past, research on psychological responses of cancer focused on the negative effects of cancer, such as its vulnerability to various mental disorders. Recently, there has been a shift to a more positive approach with emphasis on the psychological growth from the traumatic experience of having cancer [2].

Positive psychology a scientific approach to study the thoughts, feelings and behaviors of human, focusing on the good outcomes and strengths as a result of response to an adversity which allows one to progress in life instead of struggling to improve his/her life to where it was prior to the adversity [3]. Five groups of psychosocial interventions were designed to enhance positive psychology in cancer survivors, which included spiritual intervention, mindfulness-based intervention, meaning-making intervention, positive emotions expression, and hope therapy. These interventions facilitate the development of positive psychology among cancer survivors, such as benefit finding, well-being, quality of life, optimism, and hope [4–8].

One positive psychology aspect widely studied by psychologist over recent years is posttraumatic growth (PTG). PTG represents a positive psychological change experienced by one as a result of struggle arising from a life-threatening crisis or event. It has been shown to lead to an increase in personal strength, improved relationship with others, increase spiritual development, better appreciation of life, and new possibilities in life [9]. PTG has been reported in HNC patients [10, 11]. Longitudinal studies have reported stable positive changes to the trend of PTG across time from the time of diagnosis up to 18 months post diagnosis [12].

Supportive care of cancer patients covers a wide aspect of services provided by professionals who manage cancer patients. It encompasses the need for information, psychological support, social support, care for physical well-being and daily life, and spiritual needs. Unmet supportive care needs in HNC patients are common and usually arises when there is a gap between expectations and reality [13]. Psychological needs are the most prevalent unmet needs, followed by pain management, worry about treatment results, support for anxiety, change in sexual relationship, and fear of death and dying [13]. Unmet supportive care needs negatively impacts quality of life, emotional and functional adjustment, as well as the survival of cancer patients [13]. Contrastingly, fulfillment of unmet needs, particularly informational needs among HNC patients contributed to less depression and anxiety, and improved health-related quality of life [14]. As unmet supportive care needs of cancer patients exert significant negative impact on various psychological outcomes, there is an important need to investigate on how unmet supportive care needs of HNC patients affect PTG. Nevertheless, data on unmet supportive care needs and its relation to PTG in HNC patients is lacking.

Fear of cancer progression concerns about the fear and worry of cancer returning or recurring [15]. It is the most prevalent source of psychological distress among cancer survivors [16]. HNC patients have reported a high prevalence of fear of cancer recurrence ranging from 52.8% to 64.5% [17, 18]. This fear in HNC patients has been shown to be associated with a higher odd of developing anxiety and depression and act as a lifetime susceptibility risk factor for anxiety disorders or major depressive disorder [17, 18]. PTG acting as a buffering factor in reducing psychological distress and the fear of cancer recurrence has been reported in breast cancer patients [2]. However, how fear of cancer progression affects PTG in HNC patients is yet to be examined. To fill this research gap, our study aimed to: (1) investigate the level of PTG among HNC patients within the first year of cancer diagnosis and (2) determine the association between unmet supportive care needs, fear of cancer progression, and the level of PTG.

## Materials and methods

### Study design and participants

This was a multi-center cross-sectional study conducted from January 2019 to December 2019. The study population were recruited from two large oncology referral centers located in Malaysia. Sample size calculation for objective (1) was based on the formula: n = [(Z_1-α/2_ x ϭ)/Δ]^2^, in which n = total estimated sample size, Z_1-α/2_ = 95% confidence interval (critical value of 1.96 was considered), ϭ = standard deviation which was 9.0 based on the PTGI-SF score of a study of PTG in Malaysian HNC patients [6], and Δ = precision (considered as 1.5). Hence, the estimated sample size for objective (1) of the study was 138 subjects. While sample size calculation for objective (2) was based on G*Power 3.1.9.7 sample size calculator for estimating sample size for linear multiple regression and based on a study of PTG among Malaysian cancer patients [19], in which the effect size = 0.06, type I error = 0.05, power = 0.8, and the number of predictors = 12. Therefore, the estimated sample size for objective (2) was 133 subjects. Since, objective (1) required a larger estimated sample size, the estimated sample size needed for this study was 177 subjects (inclusive of an estimated dropout rate of 30%).

Consecutive sampling was employed to recruit study participants. Two research assistants who were not part of the research team were assigned for data collection on daily basis whereby HNC patients who attended the two targeted oncology referral centers were approached. Potential subjects were eligible to participate in the study if they met the following criteria: (1) diagnosis of HNC confirmed by histopathological report, (2) any stage of cancer, (3) those who were diagnosed with cancer within 1 year of duration, (4) able to read and write in the Malay language, (5) age of 18 years and above, (6) those who were physically capable of answering questionnaires, and (7) those without pre-existing history of psychiatric illnesses, such as affective disorders, anxiety disorders, psychotic disorders, illicit drug use, and alcohol use disorder (all subjects were screened with Diagnostic and Statistical Manual for Mental Disorders 5^th^ Edition by the psychiatrist in the research team). Those who met all eligibility criteria were provided with information on the purposes, procedures of the study, assurance of anonymity, and their right to withdraw from the study before they were invited to participate in the study. Written informed consent was signed before the HNC patients were enrolled in the study. This study was approved by the Human Research Ethics Committee of the two targeted referral centers.

### Measures

A self-reported questionnaire was administered to collect data on demographic and clinical characteristics. In addition, the participants were also administered the Malay versions of the Posttraumatic Growth Inventory-Short Form (PTGI-SF) to measure the degree of PTG, the 34-item Supportive Care Needs Survey (SCNS-34) to evaluate the unmet supportive care needs, and the 12-item Fear of Progression Questionnaire-Short Form (FoP-Q-SF) to estimate the fear of progression of cancer. The outcome variable in this study was posttraumatic growth. While the explanatory variables were unmet supportive care needs, fear of cancer progression, demographic and clinical characteristics.

#### Outcome variable (posttraumatic growth)

The level of posttraumatic growth among the participants was assessed with the Posttraumatic Growth Inventory-Short Form (PTGI-SF). The PTGI-SF is a self-reported instrument consisted of 10 items designated into 5 domains (new possibilities in life, relating to others, spiritual change, personal strength, and appreciation of life). Each domain comprised of two items. Each item is scored in a 6-point Likert Scale ranging from 0 to 5. Hence, the total score ranged from 0 to 50. The higher the PTGI-SF score, the higher the level of PTG in the individual being assessed. The PTGI-SF has good psychometric properties with an excellent internal consistency with a Cronbach’s α value of 0.86 [20]. The Malay version of the PTGI-SF was validated for use among Malaysian cancer patients. It demonstrated good internal consistency with a Cronbach’s α value of 0.89 [21].

#### Explanatory variables

*(1) Unmet supportive care needs*. The unmet supportive care needs among the participants in this study was measured by the 34-item self-reported Supportive Care Needs Survey (SCNS-34). It consists of 34 items designated into 5 domains (health system and information needs, patient care and support needs, psychological needs, sexuality need, and physical and daily living needs). Each item is scored in a 5-point Likert Scale ranging from 1 to 5. Each domain score is computed separately and there is no total score for the SCNS-34. The higher the domain score, the higher the level of unmet needs is for that domain. The internal consistency of its domains are excellent with Cronbach’s α ranging from 0.87 to 0.96 [22]. The Malay version of the SCNS-34 was validated among Malaysian cancer patients and all its domains exhibited good to excellent internal consistency, in which the Cronbach’s α values ranged from 0.88 to 0.93 [23].

*(2) Fear of cancer progression*. The level of fear of cancer progression and recurrence among the participants was measured by the 12-item Fear of Progression Questionnaire-Short Form (FoP-Q-SF). This instrument is a self-administered questionnaire consists of 12 items in a single domain. Each item is scored in a Likert scale of one to five ranging from 1 = never to 5 = very often. Hence, its total score ranging from 12 to 60. The FoP-Q-SF demonstrated an excellent internal consistency with a Cronbach’s α value of 0.9 [24]. The Malay version of the FoP-Q-SF was validated among Malaysian cancer patients. It demonstrated an excellent internal consistency with Cronbach’s α value of 0.93 [25].

*(3) Demographic and clinical characteristics*. The demographic characteristics data collected in this study included gender, age, religion, and monthly household income. The response for age of participants was categorized into three groups which were 18 to 40 years old, 41 to 60 years old, and more than 60 years old. Gender was recorded as male and female. Religion was registered as Islam, Buddhist, Hindu, and others. The response to monthly household income was reported as less than Malaysian Ringgit 3000, between Malaysian Ringgit 3000 to 6000, and more than Malaysian Ringgit 6000.

The clinical characteristics of the participants measured include duration from point of diagnosis, stage of cancer, the histopathological types of head and neck cancer, and mode of treatment. The answer to duration from point of diagnosis was categorized into less than 6 months, between 6 to 9 months, and more than 9 months. The histopathological types of head and neck cancer was reported as squamous cell carcinoma, adenocarcinoma, mucoepidermoid carcinoma, and others. Stage of cancer was reported as stage 1, 2, 3, and 4. Mode of treatment was reported as surgery only; chemotherapy only; surgery and radiotherapy; radiotherapy and chemotherapy; surgery and chemotherapy; and surgery, chemotherapy, and radiotherapy.

### Statistical analysis

Data analysis was performed with the Statistical Package for Social Sciences version 26 (SPSS 26; SPSS Inc., Chicago, Illinois, USA). There was no missing data. Descriptive statistics for demographic and clinical characteristics of the participants, total PTGI-SF score, total FoP-Q-SF, and SCNS-34 domain scores were performed. There was no missing data. All categorical variables (age, gender, religion, monthly household income, duration after diagnosis, type of HNC, the histopathological types of head and neck cancer, stage of cancer, and mode of treatment) were reported in frequency and percentage. As for the continuous variables (total PTGI-SF score, total FoP-Q-SF, and SCNS-34 domain scores), they were documented as mean and standard deviation (SD).

Then, a general linear model was computed to determine the association between total FoP-Q-SF and SCNS-34 domain scores (independent variables) and total PTGI-SF score (dependent variable), while controlling for the sociodemographic and clinical factors. We also checked the normality of the standardized residuals of the general linear model, which was normally distributed. Statistical significance for the general linear model was set at p < 0.05 and was two-sided.

## Results

### Participant characteristics

**Fig 1:**
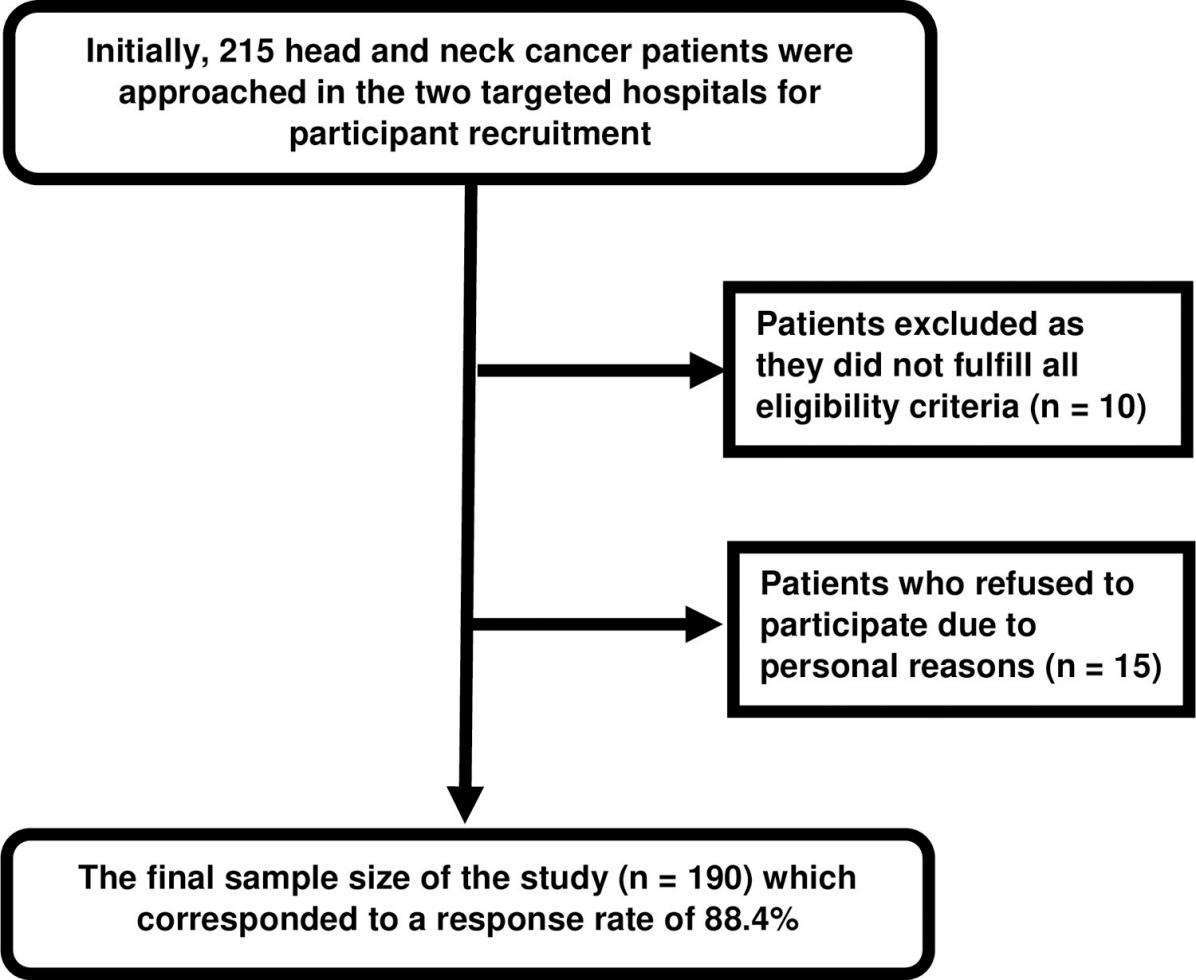
The participant recruitment process of this study.

Initially, 215 HNC patients were approached and screened for eligibility criteria. However, 10 patients were excluded as they did not fulfill all the eligibility criteria and 15 patients refused to participate due to personal reasons. Hence, the final sample size comprised of 190 participants (response rate was at 88.4%). The participant recruitment process in this study is illustrated in Fig 1.

The demographic and clinical characteristics of the participants are summarized in Table 1. Most of the participants were of middle age and there were slightly more males than females. Slightly more than three quarter of the participants were Muslims. All the participants have completed treatment they received at the time of assessment.

**Table 1:** Socio-demographic and clinical characteristics of participants.

Variables	Frequency (n)	Percentage (%)
Gender:		
Male	103	54.2
Female	87	45.8
Age:		
18 to 40 years	8	4.2
41 to 60 years	139	73.2
> 60 years	43	22.6
Monthly household income:		
< RM 3000	148	77.9
RM 3000 to RM 6000	29	15.3
> RM 6000	13	6.8
Religion:		
Islam	145	76.3
Buddhist	28	14.7
Hindu	12	6.3
Others	5	2.7
Duration since diagnosis:		
< 6 months	73	38.4
6 months to 9 months	67	35.3
> 9 months	50	26.3
Histopathological types of head and neck cancer:		
Squamous cell carcinoma	128	67.3
Adenocarcinoma	25	13.2
Mucoepidermoid carcinoma	18	9.5
Others	19	10.0
**Stage of cancer:**		
Stage 1	42	22.2
Stage 2	51	26.8
Stage 3	62	32.6
Stage 4	35	18.4
**Mode of cancer treatment:**		
Surgery only	7	3.7
Chemotherapy only	25	13.2
Surgery and chemotherapy	24	12.6
Surgery and radiotherapy	24	12.6
Chemotherapy and radiotherapy	69	36.3
Chemotherapy, radiotherapy, and surgery	41	21.6

Table 2 illustrates the total PTGI-SF, total FoP-Q-SF, and SCNS-34 domain scores. The mean of the total PTGI-SF was 39.3 (SD = 9.5).

**Table 2:** Total PTGI-SF and EORTC-QLQ-H&H-35 domain scores.

Variables	Mean	Standard deviation
**Total PTGI-SF**	39.3	9.5
**Total FoP-Q-SF**	24.1	10.8
**SCNS-34:**		
Psychological needs	23.7	10.5
Health system and information needs	23.7	12.5
Physical and daily living needs	11.5	5.6
Patient care and support needs	10.6	6.0
Sexuality needs	5.8	3.1

### The association between various factors and posttraumatic growth among the participants

Table 3 presents the general linear model of the association between total FoP-Q-SF and SCNS-34 domain scores, and total PTGI-SF score after controlling the sociodemographic and clinical factors. The model indicated that higher degree of unmet needs on physical and daily living needs was the only unmet needs domain significantly predictive of lower degree of PTG (*B* = -0.430, 95% confidence interval [CI] = -0.888 to -0.029, *t* = -1.851, p = 0.049), while higher level of fear of cancer progression also significantly contributed to lower PTG (*B* = -0.184, 95% CI = -0.323 to -0.045, *t* = -2.607, p = 0.010). None of the sociodemographic and clinical characteristics were associated with PTG among the participants.

**Table 3:** General linear model between the total FoP-SF and SCNS-34 domain scores (independent variables), and total PTGI-SF score (dependent variable) after controlling for sociodemographic and clinical factors.

Variables	*B* (95% CI)	Standard error	*t*	*p*-value
Gender:				
Male	Reference			
Female	3.037 (-0.025 to 6.098)	1.550	1.959	0.052
Age:				
18 to 40 years	Reference			
41 to 60 years	3.627 (-3.815 to 11.069)	3.768	0.963	0.337
> 60 years	5.949 (-2.199 to 14.098)	4.126	1.442	0.151
Monthly household income:				
> RM 6000	Reference			
< RM 3000	-1.947 (-8.001 to 4.108)	3.065	-0.635	0.526
RM 3000 to RM 6000	-0.722 (-7.418 to 5.975)	3.391	-0.213	0.832
Religion:				
Others	Reference			
Islam	7.258 (-1.848 to 16.364)	4.611	1.574	0.117
Buddhist	6.845 (-2.992 to 16.681)	4.981	1.374	0.171
Hindu	3.305 (-7.574 to 14.184)	5.509	0.600	0.549
Duration after diagnosis:				
< 6 months	Reference			
6 months to 9 months	-1.947 (-8.001 to 4.108)	3.065	-0.635	0.526
> 9 months	-0.722 (-7.418 to 5.975)	3.391	-0.213	0.832
Histopathological types of head and neck cancer:				
Others	Reference			
Squamous cell carcinoma	-1.897 (-6.832 to 3.038)	2.499	-0.759	0.449
Adenoma	-0.484 (-7.075 to 6.107)	3.337	-0.145	0.885
Mucoepidermoid carcinoma	-1.769 (-8.210 to 4.672)	3.261	-0.542	0.588
Stage of cancer:				
Stage 1	Reference			
Stage 2	-0.407 (-5.111 to 4.297)	2.382	-0.171	0.865
Stage 3	1.289 (-3.168 to 5.747)	2.257	0.571	0.569
Stage 4	0.370 (-3.762 to 4.503)	2.093	0.177	0.860
Mode of cancer treatment:				
Surgery only	Reference			
Chemotherapy only	-1.609 (-7.805 to 4.587)	3.137	-0.513	0.609
Surgery and chemotherapy	-4.252 (-10.440 to 1.936)	3.133	-1.357	0.177
Surgery and radiotherapy	-1.871 (-7.891 to 4.150)	3.049	-0.614	0.540
Chemotherapy and radiotherapy	-5.703 (-18.582 to 7.177)	6.521	-0.874	0.383
Chemotherapy, radiotherapy, and surgery	-3.676 (-11.130 to 3.777)	3.774	-0.974	0.331
Total FoP-Q-SF	-0.184 (-0.323 to -0.045)	0.071	-2.607	0.010*
SCNS-34:				
Psychological needs	-0.170 (-0.438 to 0.098)	0.136	-1.250	0.213
Health system and information needs	0.142 (-0.065 to 0.349)	0.105	1.357	0 .177
Physical and daily living needs	-0.430 (-0.888 to -0.029)	0.232	-1.851	0.049*
Patient care and support needs	-0.369 (-0.849 to 0.111)	0.243	-1.518	0 .131
Sexuality needs	-0.327 (-1.117 to 0.463)	0.400	-0.817	0.415

* statistical significance at *p* < 0.05

## Discussion

This multicenter study evaluated the level of PTG and the degree of unmet supportive care needs in a cohort of HNC patients within the first year from the point of diagnosis. The association between unmet supportive care needs, fear of cancer progression, and the level of PTG among HNC patients were determined after controlling for sociodemographic and clinical factors. The level of PTG in our study (mean total PTG = 39.3) was found to be comparable with that of the PTG studies among Malaysian cancer patients which also used the same instrument, the PTGI-SF (Median total PTGI-SF = 37.5; Mean total PTGI-SF = 39.87) [10, 19]. Positive psychological change has been shown to have a quadratic relationship with the point of time from diagnosis in which there is an immediate initial increasing trend before plateauing at 18 months of diagnosis [12, 26]. These findings may suggest that the trend of PTG across the time after diagnosis of cancer may be universal across different types or diagnosis of cancer. The participants in our study were within the first year from the point of diagnosis of HNC with most of them at least 6 months after diagnosis (61.6%). Due to this we expected our patients to report a high level of PTG.

Higher degree of the unmet needs regarding patients’ physical and daily living was the only type of supportive care unmet needs which predicted a lower level of PTG among HNC patients in this study, while the other types of unmet needs did not predict PTG. Unmet supportive needs in HNC required considerable attention from the treating team. Unmet supportive needs are positively correlated with anxiety and depression among HNC patients, indicating its negative impact on psychological well-being of patients [27]. Physical and daily living unmet needs are related to difficulty to cope with physical symptoms of cancer, adverse effects of treatment and managing usual tasks and activities. These include difficulty to cope with pain, fatigue or lack of energy, feeling unwell, confined to working from home and not able to perform things which patient routinely perform. Studies in HNC patients have also showed that in post-treatment period, a few issues may persist such as fatigue, sticky saliva, xerostomia, and deterioration in physical functioning. These issues may worsen the patients’ unmet physical and daily living needs [28]. Consequently, unmet needs in physical and daily living have been reported to be predictive of worrying about pain in HNC patients [29]. In addition, during the post-treatment period, HNC patients also needed more support regarding pain and worries about treatment outcomes [30]. PTG develops if the life stressor induced the meaning making process which facilitates appraisal of meaning out of the stressful event and incorporate this meaning appraisal into the core belief of the individual for reformation of a new assumption regarding self, others, and the surrounding world. However, searching for meaning will only resulting in development of PTG if a meaning is found, which facilitates psychological adaptation and allowed resolution of unproductive ruminations [2]. Unmet needs in patients’ physical and daily living may induce the search for meaning which may be highly intensive and prolonged, and failure to find meaning out of the experience of living with cancer will in turn hamper the development of PTG. Hence, the process of searching for meaning may be maladaptive and lowered the PTG among HNC participants in this study.

Our findings also highlighted the pivotal role of fear of cancer progression affecting PTG among HNC patients, whereby higher degree of fear of cancer progression contributed to lower PTG. Although searching for meaning out of the adversity of having cancer is an essential ingredient for the development of constructive PTG among cancer survivors, meaning making will not lead to development of constructive PTG which allow adaptive response to the extreme stress of having cancer, if it is unable to resolved unproductive ruminations, such as fear of cancer progression and recurrence [31]. Hence, high degree of fear of cancer progression and recurrence may lower PTG which could leave death anxiety, appraisal of cancer as threatening, uncertainty about their future medical health, and intrusive thoughts about cancer to remain unchecked [32]. Moreover, higher degree of fear of cancer recurrence also significantly associated with hopelessness, which in turn is associated with lower PTG among cancer patients [33].

Our study findings should be interpreted in the light of a few limitations. First, this study utilised a cross-sectional design and hence, we were unable to determine the causal inference of the associated factors on PTG among the participants. A prospective study is necessary to confirm the causal inference of these factors on PTG in HNC patients. Second, this study did not assess other positive psychology (hope and optimism) and social support (particularly family support) which could be important confounding factors affecting PTG among HNC patients [34, 35]. Moreover, we did not assess the effect of pain related to radiotherapy, the need for feeding tube, and other complications related to the treatment of HNC which may also be significant confounding factors influencing PTG.

Despite these limitations, this study shed some new contributions towards the understanding of the association between psychosocial issues and PTG among HNC patients. Clinically, the findings of this study alerted treating clinicians about the need to incorporate psychosocial intervention or psychotherapy which ameliorate fear of cancer progression as part of the treatment regime for HNC patients. This would enhance the development of PTG among HNC patients. For example, cognitive behavioral therapy, either as a group therapy or individual therapy, has been reported to be efficacious for reducing fear of cancer progression and recurrence [36, 37]. As our findings indicated that having higher degree of patients’ physical and daily living unmet needs contributed to lower PTG, there is also a need to further improve the psychosocial supportive care of the treating team and the supporting hospital staffs. One important point-of-focus is to generate empathy among the treating team and supportive staffs which will help to fill the gap in care for physical and daily living of patients which may be lacking in the management of HNC. There are a number of psychosocial interventions for healthcare professionals which have been evaluated and reported to improve empathy, such as communication skill training and empathy training, which employed experiential interventions (role-playing, feedback, self-awareness exercise, and group discussion with emphasis on self-learning) [38].

As recommendation for future research, it would be interesting to investigate whether positive psychology, such as hope and optimism, and social support buffer the inverse effect that fear of cancer progression had on PTG among HNC patients. Besides, it would be sensible to investigate the role of communication skill training and empathy training in enhancing PTG among HNC patients, as these interventions improve empathy among healthcare professionals.

## Conclusion

This study revealed that HNC patients within the first year after cancer diagnosis had high level of PTG. The findings highlighted that higher degree of patients’ physical and daily living unmet needs was the only type of supportive care unmet needs contributed to lower PTG, whereas the other unmet needs (such as sexuality, health system and information, patient care and support, and psychological unmet needs) were not associated with PTG. In addition, fear of cancer progression clearly contributed to lower PTG. Hence, psychosocial intervention or psychotherapy for HNC patients should emphasize on counteracting these predictors of PTG to improve the psychological well-being of patients.
